# Animal Models of Compulsive Eating Behavior

**DOI:** 10.3390/nu6104591

**Published:** 2014-10-22

**Authors:** Matteo Di Segni, Enrico Patrono, Loris Patella, Stefano Puglisi-Allegra, Rossella Ventura

**Affiliations:** 1 Dipartimento di Psicologia and Centro “Daniel Bovet”, Sapienza-Università di Roma, Piazzale Aldo Moro 5, 00181 Roma, Italy; E-Mails: matteodisegni@gmail.com (M.D.); stefano.puglisi-allegra@uniroma1.it (S.P.-A.); 2 IRCCS Fondazione Santa Lucia, via del Fosso di Fiorano 64, 00143 Roma, Italy; 3 Dipartimento di Scienze Cliniche Applicate e Biotecnologiche, University of L’Aquila, via Vetoio (Coppito 2) Coppito, 67010 L’Aquila, Italy; E-Mails: e.patrono@gmail.com (E.P.); lorispatella@gmail.com (L.P.)

**Keywords:** compulsive eating, animal models, striatum, prefrontal cortex, food addiction

## Abstract

Eating disorders are multifactorial conditions that can involve a combination of genetic, metabolic, environmental, and behavioral factors. Studies in humans and laboratory animals show that eating can also be regulated by factors unrelated to metabolic control. Several studies suggest a link between stress, access to highly palatable food, and eating disorders. Eating “comfort foods” in response to a negative emotional state, for example, suggests that some individuals overeat to self-medicate. Clinical data suggest that some individuals may develop addiction-like behaviors from consuming palatable foods. Based on this observation, “food addiction” has emerged as an area of intense scientific research. A growing body of evidence suggests that some aspects of food addiction, such as compulsive eating behavior, can be modeled in animals. Moreover, several areas of the brain, including various neurotransmitter systems, are involved in the reinforcement effects of both food and drugs, suggesting that natural and pharmacological stimuli activate similar neural systems. In addition, several recent studies have identified a putative connection between neural circuits activated in the seeking and intake of both palatable food and drugs. The development of well-characterized animal models will increase our understanding of the etiological factors of food addiction and will help identify the neural substrates involved in eating disorders such as compulsive overeating. Such models will facilitate the development and validation of targeted pharmacological therapies.

## 1. Introduction

Substance use disorders have been extensively studied in recent years, and several lines of evidence suggest that these disorders consist of neuroadaptative pathologies. Addiction is the behavioral outcome of pharmacological overstimulation and the resulting usurpation of neural mechanisms of underlying reward, motivated learning, and memory [1,2]. Although substances such as alcohol, cocaine, and nicotine are extremely popular and central to the study of addiction and substance use disorders, interest is growing in the study of compulsive activities not currently characterized as substance use disorders. One such activity is compulsive overeating [3,4,5,6,7,8].

The apparent loss of control over drug intake and compulsive drug-seeking behavior despite its negative consequences are hallmarks of drug addiction and substance use disorders [9,10,11,12]. However, addictive behaviors are not limited to drug abuse, and a growing body of evidence suggests that overeating and obesity are medical conditions that share several mechanisms and neural substrates with drug intake and compulsive drug-seeking behavior [13,14].

Drug addiction is a chronic, relapsing disorder characterized by an inability to stop or limit one’s drug intake, a strong motivation to take the drug (with activities focused on procuring and consuming the drug), and the continued use of the drug despite harmful consequences [9,12].

Many behavioral parameters of drug addiction have been recapitulated in animal models of drug addiction [9,12]. Some of these behaviors have also been reported in animal models in response to the consumption of highly palatable foods, thus introducing the notion of “food addiction” [1,7].

A scientific definition of “food addiction” has emerged in recent years, and a growing number of studies using animal models suggest that under certain circumstances, overeating can produce behavioral and physiological changes that closely resemble an addiction-like state [11,15,16,17,18].

It has been suggested that the overconsumption of so-called “refined” foods can be described as an addiction that meets the criteria used to define substance use disorders listed in the Diagnostic and Statistical Manual of Mental Disorders, Fourth Edition (DSM-IV-TR) [19,20]. Moreover, because non-drug addictions share the classical definition of addiction with substance abuse and dependence, which includes engaging in the behavior despite serious negative consequences, a new category called “Addiction and Related Behavior” was proposed by the American Psychological Association prior to the publication of DSM-V; this category should include behavioral addictions as well as addictions to natural rewards [1,7]. Finally, the Yale Food Addiction Scale was recently developed to operationalize food dependence in humans. This scale is based largely on the substance use disorders criteria defined in DSM-IV-TR, and the questions are geared specifically to the intake of highly palatable foods.

A key feature of drug addiction is compulsive use despite adverse consequences [9,10,12]; similar compulsive behavior despite negative consequences also occurs in several eating disorders including binge eating disorder, bulimia nervosa, and obesity [21]. Although there is little evidence of continued food seeking/intake despite its possible harmful consequences (an index of compulsion) in rats [22,23] and mice [24], animal models that have reproduced this behavior indicate that adaptive food seeking/intake can be transformed into a maladaptive behavior under specific experimental conditions. Based on this observation, the major goal of this paper is to review the results derived from animal models of compulsive eating behavior. Although an extended, detailed review of neurobiological and behavioral mechanisms common to drug and food addiction is beyond the scope of this paper, we also will briefly summarize some of the most important findings from studies using animal models of drug and food addiction in order to track, whenever possible, the parallels between naturally and pharmacologically rewarding stimuli.

## 2. Animal Models: Drugs of Abuse and Food

### 2.1. Animal Models

A large body of evidence suggests that generating animal models of “food addiction” is feasible, and many studies have used a palatable diet to induce overeating, obesity, binge eating, withdrawal symptoms, and food relapse in animal models [7,15,16,18,20,22,25,26,27,28,29,30,31,32,33,34,35,36,37,38,39]. In addition, one study by Avena and colleagues (2003) suggests that sugar-bingeing rats develop cross-sensitization with some drugs of abuse [40].

Although animal models cannot explain or reproduce all of the complex internal and external factors that influence eating behavior in humans, these models can enable researchers to identify the relative roles of genetic and environmental variables; this allows better control over these variables and provides for the investigation of underlying behavioral, physiological, and molecular mechanisms [11]. Animal models can be used to investigate the molecular, cellular, and neuronal processes that underlie both normal and pathological behavior patterns. Thus, animal models can advance our understanding of the many factors central to the development and expression of eating disorders.

In recent decades, the animal models in preclinical research have contributed significantly to the study of the etiology of several human psychiatric disorders, and these models have provided a useful tool for developing and validating appropriate therapeutic interventions. Inbred mouse strains are among the most commonly available and useful animal models for investigating putative gene-environment interactions in psychiatric disorders. Specifically, inbred mice have been widely used to identify the genetic basis of normal and pathological behaviors, and strain-related differences in behavior appear to be highly dependent on gene-environment interactions [41]. 

### 2.2. Compulsive Use despite Negative Consequences

#### 2.2.1. Drugs of Abuse

Many studies have investigated whether compulsive drug use in the face of adverse consequences can be observed in rodents [10,12,22]. Using intravenous self-administration (SA) of cocaine—the most common procedure for the study of voluntary drug intake in laboratory animals—Deroche-Gamonet and colleagues [22] modeled in rats some diagnostic criteria used to perform the diagnosis of addiction in humans (also see Waters *et al.* 2014 [42]):

(i) The subject has difficulty stopping drug use or limiting drug intake: the persistence of cocaine seeking during a period of signaled non-availability of cocaine has been measured.
(ii) The subject has an extremely high motivation to take the drug, with activities focused on its procurement and consumption. The authors have used a progressive-ratio schedule: the number of responses required to receive one infusion of cocaine (*i.e.*, the ratio of responding to reward) was increased progressively within the SA session.
(iii) Substance use is continued despite its harmful consequences: the persistence of the animals’ response for the drug when the drug delivery was associated with a punishment has been measured.

This study shows that, similar to addiction in humans, addiction-like behaviors in rats can be found only after a prolonged exposure to the drug. Using a “conditioned suppression” paradigm, Vanderschuren and Everitt [12] investigated whether the ability of a footshock-paired conditioned stimulus (CS) to suppress cocaine-seeking behavior diminished following a prolonged cocaine self-administration history, thus modeling compulsive drug behavior in rats. They found that cocaine seeking can be suppressed by presentation of an aversive CS, but after extended exposure to self-administered cocaine, drug seeking becomes impervious to adversity. These results indicate that an extended drug-taking history renders drug seeking impervious to environmental adversity (such as signals of punishment).

#### 2.2.2. Food

In recent years accumulating evidence suggests the possibility of modeling food addiction in animals, and different environmental conditions have been used to this end. In the “sugar addiction model” proposed by Avena and colleagues, rats are maintained on daily 12-h food deprivation, followed by 12-h access to a solution (10% sucrose or 25% glucose) and rodent chow [21,29,43,44]. After some days on this treatment, the rats show an escalation in their daily intake and binge on the solution, as measured by an increase in their intake of the solution during the first hour of access. In addition to a binge at the onset of access, the rats modify their feeding patterns by taking larger meals of sugar throughout the access period compared to control animals fed the sugar *ad libitum*. While modeling the behavioral component of food addiction, intermittent access to a sugar solution induces brain changes that are similar to the effects induced by some drugs of abuse [21,29].

In the limited access model proposed by Corwin, previous or current food deprivation is not used to induce binge-type eating, thus ruling out that the observed effects can be produced by food deprivation procedure. To provoke binge-type eating, the rats are given sporadic (generally 3 times per week), time-limited (generally 1–2 h) access to palatable food, in addition to the continuously available chow [15,45]. As described for binge eating disorder, the limited access model is able to induce binge eating in the absence of hunger [15,16,25]. Moreover, availability of addictive food (but also its shortage with periods of food restriction or dieting) are risk factors for developing eating disorders [46], and recurrent periods of caloric restriction are the strongest predictors of overeating in response to stress [47].

As discussed above, a hallmark feature of drug addiction is compulsive drug use in the face of adverse consequences [9,10,12]; similar compulsive behavior despite negative consequences also occurs in several eating disorder including binge eating disorder, bulimia nervosa, and obesity [21]. Consuming large quantities of palatable foods can indicate an increased motivation for food; however, consuming large quantities of palatable foods despite harmful consequences that result from this behavior (for example, tolerating punishment to obtain the food) is compelling evidence of a pathological food compulsion [23].

Although there is little evidence of continued food seeking/intake despite its possible harmful consequences (an index of compulsion) in rats [22,23] and mice [24], animal models that have reproduced this behavior indicate that adaptive food seeking/intake can be transformed into a maladaptive behavior under specific experimental conditions. An important key indicator of compulsive feeding is the inflexibility of the behavior, which can be assessed by temporally limiting the access to palatable food while the standard food remains available [48]. A flexible response would result in a change to available standard food, whereas an inflexible response would be revealed by neglect of the alternative, available standard food [48].

Rat models of compulsive eating have been used to study obesity and binge eating disorder [22,23,48]. To evaluate the compulsive nature of eating palatable food, these models measure the animal’s motivation to seek and consume palatable foods despite facing potentially harmful consequences. In this paradigm, negative consequences are usually modeled by pairing an unconditioned stimulus (US; e.g., a foot shock) with a conditioned stimulus (CS; e.g., light). After conditioning, the effects of exposure to the CS on palatable food seeking and consumption despite the signaled incoming punishment is measured during a test session; one can also measure the animal’s voluntary tolerance for punishment in order to obtain the palatable food. Different animal models (described below) have been proposed to assess compulsive eating behavior in the face of possible negative consequences.

(1). Johnson and Kenny [22] evaluated compulsive eating in obese male rats and found that extended access to palatable, energy-dense foods (18–23 h per day access to the cafeteria-style diet maintained for 40 consecutive days) induces compulsive-like behavior in obese rats (measured by the consumption of palatable food despite the application of a negative CS during a daily 30-min session of access in an operant chamber for 5–7 days). Moreover, they found that D2 dopamine receptors were down-regulated in the striatum of obese rats, a phenomenon that has also been reported in drug-addicted humans, supporting the presence of addiction-like neuroadaptive responses in compulsive eating.

(2). In another study, Oswald and colleagues [23] investigated whether binge eating-prone (BEP) rats, selected on the basis of a stable increase (40%) in consumption of palatable food during a 1–4 h period of time, are also prone to compulsively eating palatable foods. The heightened (*i.e.*, aberrant) motivation for palatable food was measured as the animal’s increase in voluntary tolerance for punishment in order to obtain a particular palatable food (in this case, M & M candies). Their results showed that BEP animals consumed significantly more M & Ms—and tolerated higher levels of foot shock in order to retrieve and consume those candies—than BER (binge eating-resistant) animals. This behavior emerged despite the fact that the BEP rats were sated and could choose to consume standard, shock-free chow in an adjacent arm of the maze. Together, these results confirm that BEP rats have strikingly increased motivation to consume palatable foods.

(3). Using a novel paradigm of conditioned suppression in mice, our group investigated whether a prior session of food restriction could reverse the ability of a foot shock-paired CS to suppress chocolate-seeking behavior, thus modeling food-seeking behavior in the presence of harmful consequences in mice [24].

In a recent experiment (unpublished data, [49]), we used this conditioned suppression paradigm to probe the role of gene-environment interactions in the development and expression of compulsion-like eating behaviors in mice. Thus, by modeling the inter-individual variability that characterizes clinical conditions, we found that genetic background plays a critical role in an individual’s susceptibility to develop aberrant eating behavior, thus supporting the point of view that food-related psychiatric disorders arise from a tight interaction between environmental and genetic factors.

(4). To examine the behavioral drive for dietary reinstatement after withdrawal (W), Teegarden and Bale [28] developed a reinstatement paradigm based on accessibility to the highly preferred high-fat (HF) diet in an aversive arena in mice subjected to withdrawal condition from the HF diet. In this paradigm, mice were required to endure an open, brightly lit environment to reinstate a HF diet despite the availability of house chow (less palatable food) in a less aversive setting. They found that HF-W mice spent more time on the bright side in the presence of a HF pellet in comparison with the mice in the HF non-withdrawal condition or low-fat diet control group. These results strongly demonstrated that an elevated emotional state (produced after preferred-diet reduction) provides sufficient drive to obtain a more preferred food in the face of aversive conditions, despite availability of alternative calories in the safer environment. Their data indicate that, similar to the case of an addict who is in withdrawal from a rewarding substance, mice can show risk-taking behavior to obtain a highly desirable substance.

Based on the observation that an important key indicator of compulsive feeding is the inflexibility of the behavior, Heyne and colleagues have developed a new experimental procedure to assess the inflexible nature of feeding in an animal model of compulsive food-taking behavior in rats [48]. Eating behavior has been assessed by temporally limiting the access to palatable food while the standard food was available. When rats were given a choice between standard food and a highly palatable chocolate-containing diet, they developed an inflexible food-taking behavior, as revealed by neglect of the alternative, available standard food [48].

#### 2.2.3. Withdrawal from Food

Food addiction is currently characterized by food craving, risk of relapse, withdrawal symptoms, and tolerance [7]. Two of the hallmarks of substance dependence are the emergence of withdrawal symptoms upon the discontinuation of drug use and drug craving [37]. Many different laboratories, using different animal models of food addiction (sugar-model, fat-model, and sweet-fat model [7,37]) have investigated the effects of forced abstinence from palatable food on behavior in mice and rats, by first providing animals with long-term access to palatable food and then replacing this food with standard food. However, conflicting results have been reported depending on the kind of food (sugar, fat, sweet-fat) used in different experiments [7].

Using an animal model of binge eating sugar, Avena and colleagues found that when administered the opioid antagonist naloxone, rats showed somatic signs of withdrawal [29]. Similarly, Colantuoni and colleagues [43] investigated withdrawal induced by sugar deprivation and by the administration of naloxone, which increased withdrawal symptoms (teeth chattering, forepaw tremors, head shaking) in rats fed with glucose and *ad libitum* chow, similarly to rat models of morphine addiction. Behavioral and neurochemical signs of opiate-like withdrawal have also been reported in rats with a history of binge eating sugar without the use of naloxone [50]. Moreover, a high-sugar diet has been shown to elicit signs of anxiety and hyperphagia [51], and cessation of sucrose or glucose availability induced withdrawal-like states, with increased anxiety on the plus-maze [52].

In contrast to sugar-bingeing models, withdrawal-associated symptoms have not been reported using fat-bingeing models. In fact, after 28 days on the assigned high-fat diet, spontaneous restriction and naloxone-precipitated withdrawal did not increase anxiety in the elevated plus-maze or withdrawal-induced somatic behaviors and signs of distress [17,53,54].

Finally, many studies have used a sweet-fat diet (“cafeteria-diet”) comprising diverse highly palatable foods, thus reflecting the availability and diversity of foods available to humans [7]. Using a fat-sweet diet, Teegarden and Bale [28] showed that acute withdrawal from this diet increased anxiety-like behavior, weight loss, and locomotor activity. Similar results were observed in different studies in which withdrawal from the preferred diet induced hypophagia, weight loss, and increased anxiety-like behavior in elevated plus-maze and psychomotor arousal [35,55]. Studies based on the sweet-fat diet investigated many different aspects of food withdrawal, such as the magnitude of withdrawal signs following food deprivation [56] and the role of stress and anxiety as risk factors for relapse and withdrawal symptoms [7,28].

### 2.3. Common Neurobiological Basis of Drug and Food Addiction

In addition to the above-mentioned behavioral criteria, several brain studies also support the notion that overconsumption of certain foods has several corollaries with drug addiction [54,57]. Brain areas of the reward system are involved in the reinforcement of both food and drugs through dopamine, endogenous opioid, and other neurotransmitter systems, thus suggesting that natural and pharmacological stimuli activate at least some common neural systems [58,59,60,61,62,63,64,65]. The neurocircuitry underlying food and dug addiction is complex and a review of this topic is beyond the scope of this paper. Detailed reviews of this topic can be found elsewhere [6,18,37,38,57,66].

Overall, many reviews have identified a connection between the neural circuits that are recruited while seeking/ingesting palatable food and the circuits activated while seeking/taking drugs of abuse, indicating a common profile of elevated activation in subcortical reward-related structures in response to both naturally and pharmacologically rewarding stimuli or associated cues, and a reduction in activity in cortical inhibitory regions [21,57,66,67,68]. Indeed, it appears that under different access conditions, the potent reward-inducing capacity of palatable foods can drive behavioral modification through neurochemical alterations in brain areas linked to motivation, learning, cognition, and decision making that mirror the changes induced by drug abuse [29,31,33,57,59,64,69,70]. In particular, the changes in the reward, motivation, memory, and control circuits following repeated exposure to palatable food is similar to the changes observed following repeated drug exposure [57,71]. In individuals who are vulnerable to these changes, consuming high quantities of palatable food (or drugs) can disrupt the balance between motivation, reward, learning, and control circuits, thereby increasing the reinforcing value of the palatable food (or drug) and weakening the control circuits [71,72].

#### Neurobiological Basis of Compulsion-Like Behavior

The best-established mechanism common to both food consumption and drug intake is activation of the brain’s dopaminergic reward circuitry [58,71,72]. The primary sites of these neuroadaptations are believed be the dopamine (DA), mesolimbic, and nigrostriatal circuits. The psychostimulant-induced elevation of extracellular DA levels and stimulation of DA transmission in the mesolimbic circuit is a well-known neurochemical sequence that parallels the effects of a high intake of calorie-rich palatable foods and intermittent sucrose access on activating the brain’s reward system [29,73].

Repeated stimulation of DA reward pathways is believed to trigger neurobiological adaptations in various neural circuits, thus making seeking behavior “compulsive” and leading to a loss of control over one’s intake of food or drugs [71,72]. In addition, the extent of DA release seems to be correlated with both drug-related and food-related subjective reward in humans [70,72]. Repeated stimulation of the DA system by repeated exposure to addictive drugs induces plasticity in the brain, resulting in compulsive drug intake. Similarly, repeated exposure to palatable foods in susceptible individuals can induce compulsive food consumption through the same mechanisms [29,57,64], and neuroimaging studies of obese subjects have revealed changes in the expression of DA receptors reminiscent of the changes found in drug-addicted subjects [58,64,72]. Accordingly, both cocaine addicts and obese subjects have decreased striatal D2 dopamine receptor availability, and this decrease is directly correlated with reduced neural activity in the prefrontal cortex [14,72,74]. Moreover, a growing body of evidence suggests that striatal D1 and D2 dopamine receptors (D1R, D2R) play important roles in motivated behavior [75,76,77,78,79,80,81,82].

Many factors—including the amount of effort an individual is willing to invest to receive a reward and the value that the individual places on the reward—can induce changes in motivated behavior [76,77,78,79,80], and these motivation-related factors are dependent upon dopaminergic transmission in the ventral striatum via D1R and D2R dopamine receptors. Some studies have suggested that optimal goal-directed behaviors and motivation are correlated with increased D2R expression in the striatum [80,83,84,85]. Although striatal DA transmission has been investigated extensively in recent years, the role of DA receptors in the striatum in both normal and pathological food-related motivation remains poorly understood. Nevertheless, the overconsumption of palatable foods has been shown to down-regulate dopaminergic reward circuitry through the same mechanisms that are affected in drug addiction; specifically, in humans the availability of striatal D2R dopamine receptors and DA release are reduced [71,72], leading to the hypothesis (investigated with human and animal models studies) that reduced D2R expression in the striatum is a neuroadaptive response to the overconsumption of palatable food [22,74,86,87]. On the other hand, several studies have also indicated that reduced D2R expression in the striatum may act as a causative factor, predisposing both animals and humans to overeating [22,71,87,88,89].

According to the latest hypothesis, the A1 allele of the DRD2/ANKK1 Taq1A polymorphism is strongly correlated with reduced D2R availability in the striatum, comorbid substance use disorder, obesity, and compulsive behavior [89,90]. In addition, D2R receptors were recently reported to play a critical role in ameliorating binge eating behavior in patients [6], potentially providing a target for treating some eating disorders. More studies are clearly needed to further investigate this promising therapeutic option.

Aside from the striatum, a considerable body of evidence suggests that the prefrontal cortex (PFC) plays a key role in behavioral and cognitive flexibility, as well as in motivated food-related behavior in both animals and humans [62,66,69,72,91,92]. Several areas of the PFC have been implicated in driving the motivation to eat [72,93], and several animal and human studies suggest that the PFC plays a critical role in motivated behaviors related to both food and drugs [33,58,62,69,91,92]. An abundance of data deriving from both animal and human studies suggests that PFC function is impaired in both drug addicts and food addicts [10,66,71,94]. Understanding how these dysfunctional regions in the PFC are involved in emotional processing [95] and inhibitory control [96] is particularly important for understanding addiction.

Taken together, these data show that some prefrontal regions represent a neurobiological substrate common to the drive to eat and take drugs. Functional abnormalities in these regions may enhance either drug-oriented or food-oriented behavior, depending on the established habits of the subject [58], thus leading to compulsion-like behavior.

It has been hypothesized that the transition in behavior—from initially voluntary drug use, to habitual use, and ultimately to compulsive use—represents a transition (at the neural level) in control over drug-seeking and drug-taking behaviors from the PFC to the striatum. This transition also involves a progression shift in the striatum from ventral areas to more dorsal areas, which are innervated—at least in part—by stratified dopaminergic inputs [10,97]. This progressive transition from controlled use to compulsive use seems to be correlated with a shift in the balance of behavioral control processes from the PFC to the striatum [10]. The availability of striatal D2R receptors in obese subjects is correlated with glucose metabolism in some frontal cortical areas, such as the dorsolateral PFC, which plays a role in inhibitory control [72]. Moreover, reduced dopaminergic modulation from the striatum has been suggested to impair inhibitory control over food intake and to increase risk of overeating in humans [11,71,72]. The same direct correlation between striatal D2R availability and glucose metabolism has been reported in the dorsolateral cortex of alcoholics [72].

Prefrontal DA and norepinephrine (NE) transmission has been shown to play a critical role in food-related motivation [62,71,72,98,99], as well as in the behavioral and central effects of drugs of abuse [100,101,102,103,104,105,106] in both animal models and clinical patients. Moreover, prefrontal DA and NE transmission modulate DA transmission in the nucleus accumbens under various experimental conditions [102,103,107,108,109]. In particular, altered D2R expression in the PFC has been associated with certain eating disorders and with drug addiction [14,71,72], and both α1 adrenergic receptors and D1R dopamine receptors have been suggested to play a role in regulating dopamine in the nucleus accumbens [102,103,107,108,109].

Finally, we recently investigated the role of prefrontal NE transmission in maladaptive food-related behavior in a mouse model of chocolate compulsion-like behavior [24]. Our results show that food-seeking behavior in the face of harmful consequences was prevented by selective inactivation of noradrenergic transmission, suggesting that NE in the PFC plays a critical role in maladaptive food-related behavior. These findings point to a “top-down” influence on compulsive behavior and suggest a new potential target for treating some eating disorders. Nevertheless, further research is needed in order to determine the specific role of selective prefrontal dopaminergic and noradrenergic receptors in compulsion-like eating behaviors.

### 2.4. Environmental Factors Affecting Food Addiction

Eating disorders are multifactorial conditions caused by environmental factors, genetic factors, and the complex interactions between genes and the environment [110,111]. Among the many environmental factors that can influence eating disorders such as obesity, binge eating, and bulimia, the availability of palatable foods is the most obvious [58]. The prevalence of eating disorders has increased during a time when the availability of low-cost, high-fat, high-carbohydrate foods has changed dramatically [58,112]. In fact, significant changes in the food environment have occurred and behaviors that were favored under conditions of food scarcity have become a risk factor in societies where high-energy and highly refined foods are prevalent and affordable [58]. Based on this observation, examining the addictive potential of highly processed foods has become an important goal [112,113].

In addition to quantitative aspects, the quality of the reinforcer is another critical factor for understanding food addiction and eating disorders [58]. It has been shown how different foods induce different levels of compulsive behavior [7,20,58]. In particular, palatable substances such as processed foods containing high levels of refined carbohydrates, fat, salt, and/or caffeine are hypothesized to be potentially addictive [20]. This hypothesis could explain why many people lose their ability to control their intake of such palatable foods [20]. Among palatable foods, animal studies have found that chocolate has particularly strong rewarding properties [62,114,115], as measured by both behavioral and neurochemical parameters, and chocolate is the food that is most often associated with reports of food craving in humans [116]. As a result, chocolate craving and addiction have been proposed in humans [117].

Another important environmental factor in the development and expression of eating disorders is stress. Because stress is one of the most potent environmental drivers of psychopathology, it can play a central role in eating disorders in both animals and humans [58,118,119,120,121]. Indeed, stress affects the development, course, and outcome of several psychiatric disorders, and can influence their recurrence and/or relapse after periods of remission [122,123,124,125,126,127,128,129,130]. Based on research regarding eating disorders, we now understand that stress can perturb the ability to regulate both the qualitative and quantitative aspects of food intake. Assessing stressful conditions that increase one’s susceptibility to developing an eating disorder is one of the primary goals of preclinical eating disorder research. Although both acute and chronic stress can influence food intake (as well as one’s propensity to take drugs of abuse) [58], chronic stress has been shown to increase the consumption of certain palatable foods (*i.e.*, foods that are commonly referred to as “comfort foods”) in both animals and humans [119,130,131], and chronic stress can precipitate binge eating [46,132]. Finally, several groups have reported a synergistic relationship between stress and caloric restriction in promoting the onset of eating disorders—including binge eating—in both humans and animals [11,26,27,120,121].

## 3. Conclusions

In industrialized nations, overeating is a significant problem, and overeating—particularly overeating palatable foods—leads to increased weight, obesity, and a plethora of related conditions. The continued rise in the prevalence of these conditions has prompted extensive research designed to understand their etiology, and the results of this important, ongoing research have led to policy changes in an attempt to curtail this growing problem [112].

Compulsive eating despite negative consequences is prevalent among patients who suffer from eating disorders such as bulimia nervosa, binge eating disorder, and obesity. Moreover, this behavior is strikingly similar to the phenomenon observed in individuals with compulsive drug-seeking/intake behavior. Because the increasingly compulsive use of drugs in the face of well-known detrimental consequences is a classic behavioral feature of drug addiction, it has been suggested that compulsive overeating—particularly overeating of refined foods—should be classified as a bona fide addiction (*i.e.*, “food addiction”). Indeed, such behavior satisfies the DSM-IV-TR diagnostic criteria for substance use disorders [20], and the Yale Food Addiction Scale, which is currently the most widely used and accepted tool for measuring food addiction [7], was recently developed to operationalize the construct of food addiction, adapting DSM-IV-TR criteria for substance dependence as applied to food [66]. Although these criteria are also present in the new edition of the DSM V (the most recent edition [133]), suggesting that non-substance-related disorders are related to the use of other rewarding stimuli (*i.e.*, gambling), the DSM V does not categorize similar disorders related to natural rewards as behavioral addictions or substance use disorders [7].

Moreover, the literature indicates that food craving frequently results in binge episodes, during which a greater-than-normal quantity of food is ingested in a shorter-than-normal period of time. Importantly, the prevalence of bingeing increases with the body mass index (BMI) and more than one-third of binge-eaters are obese [15]. However, binge eating disorder and food addiction are not correlated with BMI and high BMI is not a predictive factor of compulsive eating [86]. Obesity is a possible, but not obligatory result of compulsive behavior towards food; although the indices of obesity measured by BMI often correlate positively with the index of food addiction measured by the YFAS, they are not synonymous [3,66,134]. This dissociation has been modeled in pre-clinical studies that demonstrate that the development of fat bingeing behavior is not associated with weight gain, supporting the idea that obesity and food addiction are not reciprocal conditions [25,135].

Stressful life events and negative reinforcement can interact with genetic factors, thereby increasing the risk of addictive behaviors and/or inducing changes in the corticostriatal dopaminergic and noradrenergic signals involved in motivational salience attribution processes [62,107,109]. Inbred mouse strains are a fundamental tool for performing genetics studies, and studies comparing different inbred strains have yielded insight into the role that genetic background plays in the dopaminergic system in the midbrain and dopamine-associated behavioral responses [107]. Although they are desperately needed, however, studies of gene-environment interactions in human eating disorders are extremely rare [110]; to date only a handful of animal studies have investigated the specific role of the interaction between environmental factors and genetic factors in the development and expression of compulsive food seeking/intake despite harmful consequences (*i.e.*, an index of compulsion) in rats and mice [22,23,48,136].

Our preliminary data (data not shown, [49]) indicate that compulsive eating emerges following extended access to a highly palatable diet [22], similar to how compulsive drug seeking emerges following an extended history of drug-taking [9,12], but only in genetically susceptible subjects.

Developing well-characterized and validated animal models of compulsive overeating will provide an essential tool for advancing our understanding of the genetic and behavioral factors underlying eating disorders. In addition, these models will facilitate the identification of putative therapeutic targets and help researchers develop, test, and refine suitable pharmacological and cognitive behavioral therapies.
